# Effect of a Short Time Concentric Versus Eccentric Training Program on Electromyography Activity and Peak Torque of Quadriceps

**DOI:** 10.2478/hukin-2014-0027

**Published:** 2014-07-08

**Authors:** Alberto Carvalho, Paolo Caserotti, Carlos Carvalho, Eduardo Abade, Jaime Sampaio

**Affiliations:** 1 Research Center of Sports Science, Health and Human Development (CIDESD).; 2 High Institute of Maia (ISMAI), Portugal.; 3 University of Trás-os-Montes e Alto Douro (UTAD), Portugal.; 4 University of Southern Denmark, Odense, Denmark.

**Keywords:** Isokinetic training, quadriceps, hamstring, peak torque, imbalance ratios

## Abstract

The purpose of this study was to examine the effect of an 8-week concentric (CON) versus eccentric (ECC) isokinetic training program on the electromyography (EMG) signal amplitude of vastus medialis (VM), vastus lateralis (VL) and rectus femoris (RF). Also, the isometric (ISO) and dynamic maximum strength of the knee extensors were assessed. Eighteen physically healthy male subjects (age 22 ± 1 years, body height 177 ± 4 cm, body mass 73 ± 7 kg) performed four weeks of unilateral CON isokinetic training for the quadriceps of the dominant leg on a REV9000 dynamometer. At the end of the fourth week, the sample was divided into two groups, with one group performing additional four weeks of unilateral ECC training and the other continuing with CON training. The training sessions consisted of three sets of ten maximal repetitions at a velocity of 60ºs-1, three days per week for eight weeks. The results showed that CON and ECC groups improved the peak torque in all types of contractions. Also, both groups presented increases in the avgEMG for VL, VM and RF. The present investigation showed that CON training elicited increases of the ISO peak torque and VM avgEMG in the CON contraction. Additionally, significant gains were reported after the ECC training on the VM avgEMG in all contractions and RM avgEMG in CON contraction.

## Introduction

Strength training programs are based on exercises performed in dynamic conditions involving both concentric (CON) and eccentric (ECC) muscle actions. Isokinetic evaluations have progressively been increasing in scientific research and sports medicine and are considered a reliable method to test and measure dynamic strength (L. E. Brown and Weir, 2001; Capranica et al., 1998; Croisier et al., 2003; Evetovich et al., 1998). In fact, isokinetic data are used in sports training to predict and improve the players’ performance (Aagaard et al., 1995; Li et al., 1996). Short-term isokinetic training programs may be useful to increase the strength and muscular performance (Beckerman et al., 2001; Kaminski et al., 1998), while having strong implications in physical medicine therapies, design of rehabilitation programs and improvements in sports performance.

Several studies have shown significant improvements in ISO and dynamic strength after short-term isokinetic training programs. For example, increases in the isokinetic peak torque (PT) of the leg extensors were already reported after two (Prevost et al., 1999) and three training sessions (Coburn et al., 2006). Also, short-term improvements were found in the rate of velocity development during maximal CON isokinetic leg extensions after two training sessions (L. Brown and Whitehurst, 2003). Moreover, gains in the ECC mode were reported after a 2-week program (3 sessions per week) that consisted of 5 sets of 8 knee extensions (Brassine et al., 2002). Yet, other investigations have found no differences in strength gains of upper and lower extremity muscles between ECC and CON training programs (Johnson, 1972; Pavone and Moffat, 1985). Nevertheless, ECC training seems to be more efficient than CON (Asmussen, 1953; Komi, 1973). In fact, several studies have reported more specific and effective strength gains after ECC training in maximal voluntary strength (Higbie et al., 1996; Kaminski et al., 1998; Roig et al., 2009; Seger and Thorstensson, 2005).

Previous research has suggested that strength gains have a strong correlation with the training mode i.e. greater CON strength gains obtained after CON training and greater ECC gains after ECC training (Duncan et al., 1989; Tomberlin et al., 1991). During a resistance-training program, increases in muscular strength are commonly attributed to two main factors: a) neural adaptations, such as increased activation of agonist and/or synergist muscles involved in the muscle action, improved coordination and reduced co-activation of antagonist muscles, and b) increases in muscle fiber size (hypertrophy) (Moritani and Devries, 1979).

It has already been reported that neural factors have a strong influence on the strength gains during the first 3 weeks of a resistance-training program. On the other hand, hypertrophy methodologies seem to be more effective in increasing strength gains after 3 to 5 weeks of training (Hakkinen et al., 1981; Moritani and Devries, 1979). Although skeletal muscle adaptations may contribute to strength gains (Staron et al., 1994), the available literature suggests that neural adaptations may be particularly important during the early phases of a resistance-training program (Amiridis et al., 1996; Holtermann et al., 2005; Moritani and Devries, 1979). As a consequence, the improvements in strength and muscular performance that occur immediately after a short-term training program are not likely to be attributed to muscle fiber hypertrophy (Prevost et al., 1999) but mainly to neural factors.

The assessment of neural adaptations has been frequently performed with surface electromyography (EMG), a common technique for examining muscle function in kinesiology, exercise physiology and biomechanics (Aboodarda et al., 2011; van den Tillaar and Ettema, 2013).

The surface EMG signal comprises the sum of the electrical contributions generated by the active motor units (Farina et al., 2004). Thus, it is suggested that EMG amplitude is primarily influenced by the level of the muscle activation that depends on the number of active motor units and their firing rates (De Luca, 1997). Although the inherent nature of the signal implies some limitations related to the interpretation of the surface EMG amplitude and frequency data (Farina et al., 2004), EMG is considered a valid tool for the investigation of neuromuscular adaptations in resistance training (Akima et al., 1999; Garfinkel and Cafarelli, 1992; Hakkinen et al., 1998; Holtermann et al., 2005). In fact, a previous research focused on the post activation mechanism has considered that the lack of electromyographic recordings was a limitation when evaluating the power output production (Liossis et al., 2013).

The processing methodology about the behaviour of the signal in time domain, is described by amplitude variables that are related to the root mean square of the signal or the average rectified value and the muscle fiber conduction velocity (Clancy, 1999; De Luca, 1997). Neural adaptations contribute to the increase in muscular strength in both CON and ECC isokinetic training programs, yet, gains seem to be higher after ECC training.

The selection of adequate types of muscular contractions (ECC and CON) is a very important issue to strength and conditioning coaches (Krol and Mynarski, 2012). So, it is vital that scientific research compares and describes with accuracy the effects of both types of contraction on sports performance (Drury et al., 2006). Therefore, the purpose of this study was to examine the effects of an 8-week CON versus ECC isokinetic training program on the electromyogram signal amplitude, ISO and dynamic maximum strength in the knee extensors.

## Material and Methods

### Subjects

Eighteen physically healthy subjects (mean age ± SD: 22 ± 1 years, body height: 177 ± 4 cm, body mass: 73 ± 7 kg) with no cardiovascular or orthopaedic problems and without previous experience with isokinetic tests volunteered to participate in this study. After four weeks of CON training, the sample was divided into two groups: one group performed additional four weeks of unilateral ECC training (n=9; ECC group, age 22 ± 1 years, body height: 177 ± 4 cm, body mass: 72 ± 6 kg) and the other continued with CON training (n=9; CON group, age 22 ± 1 years, body height: 177 ± 4 cm, body mass: 75 ± 8 kg). Prior to the study, a disclosure document was presented to the players indicating all the associated procedures, benefits and potential risks. A written consent form in accordance with the latest revision of the Declaration of Helsinki was also required so that this protocol would be approved by the ethics committee of the Research Centre in Sports Sciences, Health and Human Development (Portugal).

### Procedures

All sessions were carried out at the same time, more than 3 hours after a meal with the room temperature maintained at 21ºC. All sessions were preceded by a 10 minute warm-up on an ergocycle (70 rpm at 50 watts) and dynamic stretching exercises (for hamstrings and quadriceps). After that, a 5 minute interval was conceded for the electrodes placement and another 5 minutes for the isokinetic evaluation of each leg. Accordingly to the available sample and in order to maintain the schedule, six participants were evaluated in each session. A similar ECC/CON ratio criterion was used to place subjects in each group (9) (CON group, 1,27 ± 0,22; ECC group, 1,37 ± 0,16 p = 0,270).

Subjects sat with their thighs at an angle of 85*º* to the trunk. The mechanical axis of the dynamometer was aligned with the lateral epicondyle of the knee. The trunk and thighs were stabilized with belts. The knee range of motion was 70º (20 to 90º of flexion); the lever arm was positioned at the distal third of the leg; torque was gravity-corrected and dynamometer calibration was performed before every session in accordance with the manufacturers’ instructions. All sessions began with the assessment of the right lower limb and proceeded in the same order: 5 CON contractions at 60ºs^−1^ followed by ISO muscle contractions (flexion and extension) during 5 s (at 60º of flexion) and 5 ECC contractions at 60ºs^−1^.

A 90 s recovery period was allowed between CON, ISO and ECC tests. All subjects benefited from both visual feedback and verbal encouragement. The highest value for each testing condition was registered for further analysis. All procedures to assess muscular strength and power (L. E. Brown and Weir, 2001) were based on the recommendations of the American Society of Exercise Physiology. All tests were performed with the use of the REV9000 (Technogym, Italy) isokinetic dynamometer and controlled by the same qualified technician. EMG signals of *Vastus Lateralis* (VL), *Rectus Femoris* (RF) and *Vastus Medialis* (VM) muscles were recorded in all subjects using surface electrodes (Medicotest, Blue Sensor) with bi-polar placement according to SENIAM’s recommendations for surface EMG. The data were analysed considering AvgEMG of each muscle. For the RF evaluation, the electrodes were placed at 50% on the line from the anterior spina-iliaca superior to the superior part of the patella. The electrodes were orientated in the direction of the line from the anterior spina iliaca superior to the superior part of the patella. For the VM, the electrodes were placed at 80% on the line between the anterior spina iliaca superior and the joint space in front of the anterior border of the medial ligament. The orientation was almost perpendicular to the line between the anterior spina iliaca superior and the joint space in front of the anterior border of the medial ligament. Finally, for the VL, the electrodes were placed at 2/3 on the line from the anterior spina iliaca superior to the lateral side of the patella. The orientation was in the direction of the muscle fibers. In all cases an elastic band was used to the fixation on the skin.

To evaluate the muscle electrical activity, a pre-amplified telemetry system (Glonner) was used with *Simi Motion* software. For all muscles normalized amplitudes were obtained by calculating the percentage of the average EMG full signal amplitude of the movement with respect to the maximal isometric action (Table 1). The signal was recorded at 2000 Hz and smoothed using a low-pass 10 Hz filter. EMG Signal analysis was performed through a window of 0,512 s centred in the force curve produced in the ECC and CON contractions. For ISO contractions a 2 s window centred in the force curve was used.

### Isokinetic training program

The 8-week training program included three sessions per week, which resulted in a total of 24 sessions. The isokinetic program was performed in 3 sets of 10 maximum repetitions to both flexor and extensor muscle groups of the dominant leg. The non-dominant leg was not involved in training.

The subjects performed a five minute warm-up on a cycle ergometer (75 W) and dynamic stretching exercises for quadriceps and hamstring muscles of the trained leg (3 × 10 repetitions with a 30 s rest interval).

After that, the subjects seated on the isokinetic dynamometer REV9000 (Technogym, Italy) with the backrest reclined 5º from vertical and straps fixing the trunk, waist and distal thigh. The lateral femoral epicondyle was used as the body landmark for matching the rotation axes of the knee joint and the lever arm of the dynamometer. The dynamometer pad was then fastened around the leg 5 cm proximally to the medial malleolus, and the subjects performed a series of three submaximal contractions for familiarization. Then, the participants performed three series of ten consecutive maximal ECC isokinetic contractions; the knee was moved by the dynamometer through the range of motion from 20 to 90º of knee flexion at an angular velocity of 60ºs^−1^. Each series was preceded by three minutes of rest and no pauses were used between the ten contractions.

### Statistical Analysis

Mean and standard deviations were calculated for AvgEMG signals of *Vastus Lateralis* (VL), *Rectus Femoris* (RF), *Vastus Medialis* (VM) and peak torque of all data. Detection of systematic biases was performed using a repeated measures *t*-test. Independent samples *t*-test was used to seek differences between CON and ECC groups (9). The probability of type I error (alpha) was set a priori at 0.05 in all statistical analyses. All statistical procedures were performed with SPSS 17.0 statistical software (SPSS Inc., Chicago, IL, USA).

## Results

Table 1 presents the mean, standard deviation and percentage differences of avgEMG (mV) in VL, RF and VM muscles and quadriceps peak torque (Nm) before and after CON and ECC isokinetic training.

The CON group increased 6, 7 and 7% in CON, ISO and ECC peak torques, respectively. The increases of VL avgEMG were of 9% in CON, 7% in ISO and 9% in ECC contractions. For the RF, the increases of avgEMG were of 5% in CON, 2% in ISO and 5% in ECC contractions. For VM, the increases of avgEMG were higher in CON (9%) and ISO (10%) than in ECC contractions (5%).

The ECC group improvements were higher in ECC (10%), than in ISO (6%) and CON (1%) peak torques. For VL, the increases of avgEMG were higher in CON (17%) than in ISO (14%) and ECC contractions (15%). For the RF, the increases of avgEMG were similar in CON (8%), ISO (3%) than in ECC (4%) contractions. The increases of the VM avgEMG were of 7% in ECC, 10% in ISO and 11% in CON contractions.

## Discussion

The purpose of this study was to examine the effect of an 8-week CON versus ECC isokinetic training on the electromyogram signal amplitude (EMG), ISO and dynamic maximum strength in the knee extensors. The results showed that an 8-week isokinetic training program (3 days per week) with 3 sets of both 10 CON or ECC maximum knee extensions is effective in increasing muscle activation and maximum voluntary contraction of ISO and dynamic strength of the knee extensors.

Strength gains after CON and ECC training are highly dependent on the muscle action used for training and testing. Muscle hypertrophy and neural adaptations contribute to strength increases in both CON and ECC training (Higbie et al., 1996). It was already found that the effects of unilateral CON isokinetic leg extension training over twelve weeks resulted in a significant increase of peak torque in the training group and no significant changes in the control group (Evetovich et al., 1998). The increases in peak torque were suggested by the authors to be related to hypertrophy and/or changes in other muscles involved in leg extension. Another study found a significant increase of knee extensor torque in both limbs (right, from 229 ± 54 to 304 ± 53 Nm; left, from 228 ± 59 to 311 ± 63 Nm), after six weeks of bilateral ECC isokinetic training (at 30ºs^−1^), of the knee extensors (Poletto et al., 2008).

When compared to CON programs, ECC training appears to be more specific and effective in maximal voluntary strength. A previous investigation showed that the CON group improved by 19% while the ECC group improved by 29% after 6-weeks of CON or ECC hamstring strength training (Kaminski et al., 1998). Another investigation that compared the effects of 10 weeks of CON *vs.* ECC isokinetic training on quadriceps muscle strength, showed that ECC muscle actions after ECC training was significantly greater than the corresponding change in average torque measured during CON muscle actions after CON training (36,2% *vs.* 18,4%) (Higbie et al., 1996).

This trend was also confirmed in a study that compared ECC *vs.* CON exercise in stimulating gains and muscle strength which found that ECC training performed at high intensities was more effective in promoting increases in muscle mass measured by muscle girth (Roig et al., 2009).

The efficiency of ECC training in increasing muscle strength and mass appears to be related to the higher loads developed during ECC contractions. An investigation that compared ECC and CON unilateral isokinetic knee extensor (4 sets of 10 maximal efforts at 90ºs^−1^, 3 days a week) concluded that changes in strength revealed more signs of specificity related to velocity and contraction type after ECC than CON training (Seger and Thorstensson, 2005).

The present results also show that ECC training induces a greater mode-specific stimulus for an increase in strength because it augments ECC strength more than CON training increases CON strength. The gains in the ECC group were higher than the CON group in ECC strength (10% *vs.*7%) and lower in CON strength (1% *vs.* 6%) compared with CON training. The CON group had similar gains in CON and ECC maximal strength.

Gains in muscular strength after CON and ECC isokinetic training are highly dependent on the muscle action used for training and testing. ECC is more effective than CON isokinetic training for developing strength in ECC isokinetic muscle actions, and CON is more effective than ECC isokinetic training for developing strength in CON isokinetic muscle actions.

Literature has already compared the effect of a 10-week resistance-training program on peak ISO torque, muscle hypertrophy, voluntary activation and electromyogram signal amplitude (EMG) of the knee extensors between young and elderly women. Similar increases in peak ISO torque (16% and 18%) and EMG (19% and 21%) were observed between young and elderly women (Cannon et al., 2007). These findings provide evidence to indicate that participation in regular resistance programs may induce significant neuromuscular benefits in women independent of age. However, another study found that maximal strength gains are affected by age. The results showed that young subjects had a greater increase (34%) than older subjects (28%) after 9 weeks of unilateral knee extension strength training (3 days a week) and there were no significant differences in strength gains between men and women in either age group (Lemmer et al., 2000).

Even short-time strength training programs show significant improvements in ISO and dynamic strength of the knee extensors. For instance, a 22,1% increase was already reported in isokinetic leg extension peak torque after just 2 training sessions (Prevost et al., 1999). In another study, the effects of 2 training sessions resulted in significant improvements in the rate of velocity development during maximal CON isokinetic leg extensions (L. Brown and Whitehurst, 2003). In addition, significant increases in isokinetic leg extension peak torque were already found after just 3 training sessions (Coburn et al., 2006). All of these short-term gains are probably linked to neural, rather than muscle structural adaptations (Prevost et al., 1999).

A short-time training program that consisted of 5 sets of 8 knee extensions in ECC mode, 3 times a week, during 2 weeks, found about 21% of gains in ECC mode and no differences were found in CON and ISO modes. In EMG activity of VL, VM and RF all muscles increased their activity in pre-post comparison, but not significantly (Brassine et al., 2002). These findings support the idea of mode-specificity training and suggest that short-term isokinetic training may not be sufficient to have significant increases in EMG activity. In fact, an earlier investigation found no significant differences in forearm flexion and extension torque and EMG amplitude for the agonist and antagonist muscles after 2 days of isokinetic training (Beck et al., 2007). Another study concluded that there were no pre-test to post-test changes in EMG amplitude after 3 days of slow or fast velocities (30ºs^−1^ and 270ºs^−1^) isokinetic training (Coburn et al., 2006). After that protocol, changes were only reported in muscular strength: the slow velocity group had an increase of 24,4% at 30ºs^−1^ and 11,5% at 270ºs^−1^ in PT, while the fast velocity group increased 40,2% in PT at 270ºs^−1^ only. The control group had no changes in PT at either velocity.

Additionally, the effects of 10 weeks CON *vs.* ECC isokinetic training on quadriceps muscle strength, cross-sectional area and neural activation were compared (Higbie et al., 1996). No significant differences were observed in the changes of maximal iEMG between the ECC and CON group. When tested in the ECC mode, the percentage changes in maximal iEMG were 16,7% for the ECC and 20,0% for CON group. When tested in the CON mode, the percentage changes in maximal iEMG were 7,1% for the ECC and 21,7% for CON group.

Our investigation shows clear signs of specificity in ECC training, since the percentage gains of avgEMG in VL of the ECC group were higher than the CON group in CON (17% *vs.* 9%), ISO (14% *vs.* 7%) and ECC contractions (15% *vs.* 9%). In VM, the percentage gains of avgEMG of the ECC group were higher in CON (11% vs 9%) and ECC (7% vs 5%) contractions and identical in ISO (10% vs 10%). In RF, the percentage gains of avgEMG were higher in the ECC group for CON (8% *vs.* 5%) and ISO (3% *vs.* 2%) and the same in ECC (5% vs 5%) contractions. The neural adaptations contribute to strength increases after CON and ECC isokinetic training, however, it should be noticed that neural adaptations seem to be more significant in ECC training, probably because of the higher loads experienced during ECC contractions.

In conclusion, this study showed that CON training improved both peak torque and electrical activation in all types of contractions (CON, ISO and ECC), mainly in avgEMG *of Vastus Medialis* and *Vastus Lateralis* muscles. Comparatively to the CON training, the ECC training significantly improved the electrical activation in all muscles. Also, the avgEMG of both *Vastus Medialis* and *Vastus Lateralis* muscles obtained higher values compared to the avgEMG of *Rectus Femoris* in all types of contraction.

## Figures and Tables

**Table 1:** Normalized values of the average EMG (%) full signal amplitude in VL, RF and VM muscles and knee extensors peak torque (Nm) before and after concentric vs. eccentric isokinetic training

			CONCENTRIC GROUP (CON)			ECCENTRIC GROUP (ECC)		
	
			Pre-test	Post-test	Dif.	Pre-test	Post-test	Dif.
MUSCLE STRENGHT ASSESSEMENT	CONCENTRIC	VL	21,42 ± 7,17	30,71 ± 18,19	9%	21,10 ± 7,17	37,99 ± 16,81	17%
		RF	20,45 ± 9,19	25,30 ± 18,77	5%	16,91 ± 3,91	24,68 ± 11,60	8%
		VM	20,63 ± 5,41	29,29 ± 15,44	9%	21,16 ± 6,21	31,79 ± 10,77	11%
		PT	209,25 ± 32,36	221,63 ± 24,87	6%	210,25 ± 25,50	212,50 ± 33,83	1%
	ISOMETRIC	VL	18,99 ± 6,99	26,29 ± 13,58	7%	14,89 ± 4,97	28,57 ± 17,77	14%
		RF	14,49 ± 4,42	16,79 ± 8,25	2%	14,40 ± 2,42	18,28 ± 4,18	3%
		VM	15,19 ± 5,47	24,72 ± 11,02	10%	15,85 ± 4,20	25,77 ± 13,10	10%
		PT	232,13 ± 46,70	248,35 ± 43,09	7%	238,40 ± 44,14	252,13 ± 46,08	6%
	ECCENTRIC	VL	20,17 ± 6,44	28,84 ± 16,14	9%	16,81 ± 5,63	31,78 ± 19,48	15%
		RF	15,31 ± 5,95	20,05 ± 12,01	5%	15,87 ± 4,61	20,45 ± 8,69	5%
		VM	18,48 ± 3,59	23,40± 11,67	5%	19,63 ± 7,00	26,66 ± 11,21	7%
		PT	270,56 ± 63,66	291,56 ± 87,08	7%	284,22 ± 63,23	314,11 ± 72,56	10%
